# Psychosocial correlates of performance-enhancing substance use intentions and prevention needs among recreational gym users: a theory-informed mixed-methods study

**DOI:** 10.1186/s40359-026-05476-4

**Published:** 2026-08-29

**Authors:** René Paasch, Gunnar Mau

**Affiliations:** 1 Department of Psychology, German University of Health and Sport, Vulkanstraße 1, 10367 Berlin, Germany; 2 Department of Marketing and Retailing, Magdeburg-Stendal University of Applied Sciences, Breitscheidstr. 2, 39114 Magdeburg, Germany

## Abstract

**Background:**

Performance-enhancing substance (PES) use is a potential public health concern in recreational fitness. Research suggests that use among recreational gym users is associated with psychosocial factors, gym culture, and peer norms.

**Objective:**

This study examined associations of Theory of Planned Behavior (TPB) constructs, selected Health Belief Model (HBM) constructs, knowledge, and risk acceptance with PES-use intentions and explored prevention needs using a mixed-methods design.

**Methods:**

A cross-sectional mixed-methods survey included 772 recreational gym users in Germany; 29.4% reported ever or current use of at least one of six assessed PES categories. Quantitative analyses examined TPB constructs, HBM-related constructs, knowledge, and risk acceptance. Qualitative data comprised 542 open-ended responses on prevention preferences.

**Results:**

Subjective norms and lower perceived behavioral control over avoiding PES showed the strongest associations with higher PES-use intention. Greater knowledge was associated with lower intention, whereas higher risk acceptance, greater perceived benefits of PES use, and lower self-efficacy to resist PES were associated with higher intention. None of the examined psychosocial variables were significantly associated with the ever/current PES-use indicator. Qualitative findings emphasized credible information, peer-based support, and preferences for gym-based prevention approaches.

**Conclusion:**

Psychosocial factors were associated with variation in PES-use intentions among recreational gym users. Prevention development may consider social norms, perceived control over avoiding PES, resistance self-efficacy, knowledge, and preferences for credible, context-sensitive information. Given the cross-sectional design, the findings should be interpreted as associations and as hypothesis-generating evidence for future prevention research.

**Supplementary information:**

The online version contains supplementary material available at https://doi.org/10.1186/s40359-026-05476-4.

## Introduction

The nonmedical use of image- and performance-enhancing drugs (IPEDs), referred to throughout this article as performance-enhancing substances (PES) for readability, including anabolic–androgenic steroids (AAS), growth hormone (GH), and testosterone replacement therapy (TRT), is increasingly recognized as a potential public health concern in recreational fitness contexts. Although these substances have traditionally been associated with elite sport, empirical research indicates that nonmedical PES use also occurs among recreational gym users, often in social environments shaped by appearance- and performance-related norms [1]. International studies suggest that such use may be embedded in specific gym cultures and lifestyle practices, where peer norms, bodily enhancement goals, and performance-oriented expectations are associated with substance-related decision-making [2, 3]. These dynamics have been linked to continuous striving for bodily enhancement, sometimes described as a “hedonic treadmill” of appearance- and performance-oriented goals [4, 5].

In the present study, PES refers to the nonmedical use of substances intended to enhance appearance, muscularity, performance, or recovery. The assessed categories comprised AAS, growth hormone, TRT/testosterone, fat burners, stimulants, and boosters. The latter three categories were assessed broadly without product-level definitions and may therefore have encompassed legally available products. Conventional nutritional supplements such as protein powders or creatine were not assessed as PES categories, although supplement use has been associated with increased susceptibility to illicit substance use [6]. Meta-analytic estimates indicate lifetime AAS prevalence rates of approximately 3–6% in recreational populations [1], with substantially higher rates reported in specific gym-based samples [7]. Documented health risks associated with nonmedical use include cardiovascular complications, endocrine disruption, psychological distress, body image pathology, and dependency-related symptoms [8]. Importantly, PES-related behaviors have been associated not only with individual psychological factors but also with social and structural factors such as peer norms, availability, and gym culture [9]. In the present sample, 29.4% reported ever or current use of at least one of the six assessed PES categories, indicating that PES-related behavior was present in a substantial proportion of the sample.

Despite these risks, prevention efforts targeting PES use remain heterogeneous and are often not explicitly grounded in behavioral theory, particularly outside elite sport settings [10–12]. A recent meta-analysis reported generally small effects for existing prevention programs and noted that most evaluated interventions were conducted in competitive athlete populations, limiting their generalizability to recreational gym users [13]. In Germany, theory-informed evidence on psychosocial factors associated with PES use and prevention needs among recreational gym users remains limited. Further empirical research in this population is therefore needed.

The present study adopts the Theory of Planned Behavior (TPB) [14] as its primary theoretical framework for examining PES-related intentions among recreational gym users. Given that PES-related decisions involve not only motivational but also health-related evaluations, selected constructs from the Health Belief Model (HBM) [15, 16] were included as complementary health-related constructs. In addition, doping-related knowledge and risk acceptance were considered as theoretically informed supplementary variables, as both have been discussed as relevant factors in performance-enhancing substance use research. A more detailed rationale for this theoretical integration is provided in the following section.

The primary aim of the present study was to examine associations between these psychosocial factors and PES-use intentions among recreational gym users. A cross-sectional mixed-methods design with a sequential explanatory analytical logic combined quantitative analyses with a qualitative exploration of prevention needs derived from open-ended responses (*n* = 542). By integrating both strands, the study sought to provide a more comprehensive account of factors associated with PES-use intentions and to inform hypotheses for prevention development in recreational fitness settings.

## Theoretical framework

Understanding PES use in recreational fitness requires consideration of both intentional decision-making and health-related risk evaluations. The Theory of Planned Behavior (TPB) served as the primary theoretical framework. Selected Health Belief Model (HBM) constructs, together with knowledge and risk acceptance, were included as complementary components.

### Theory of planned behavior (TPB)

The TPB has been widely applied to performance-enhancing substance use and other health-risk behaviors. Attitudes, subjective norms, and perceived behavioral control (PBC) have shown consistent associations with behavioral intentions, whereas associations with behavior have been less consistent [12, 17, 18]. Although the TPB does not fully capture all factors associated with health-risk behavior, it remains a parsimonious and empirically supported framework for examining behavioral intentions when its assumptions and boundaries are clearly specified [19]. Accordingly, the TPB was adopted as the primary theoretical framework for examining PES-use intention in the present study. In the present study, however, PBC was operationalized specifically as perceived control over avoiding or abstaining from PES, rather than as perceived control over PES use itself.

### Health belief model (HBM)

Selected HBM constructs [15, 16] were included to complement the TPB with a health-oriented perspective. Rather than applying the full HBM, the study focused on constructs relevant to risk and cost-benefit evaluations of PES use. Perceived health risk and severity-related evaluations were combined into an index of risk perception, reflecting an overall appraisal of potential health harms. This approach is consistent with previous research suggesting that these dimensions may operate jointly in substance-related decision-making [10].

Perceived benefits of PES use, perceived barriers to abstaining from PES, and self-efficacy to resist PES use were also assessed. Higher risk perception was expected to be associated with lower PES-use intention, whereas greater perceived benefits of PES use and greater perceived barriers to abstaining were expected to be associated with higher intention. Greater self-efficacy to resist PES use was expected to be associated with lower intention and a lower likelihood of ever/current PES use.

For the statistical analyses, the PBC and self-efficacy composites were reverse coded. Consequently, higher analytical scores represent lower perceived control over avoiding PES and lower self-efficacy to resist PES, respectively. Importantly, reverse coding changed only the direction of the analytical scores and did not alter the behavioral target of the underlying items. The PBC measure therefore reflects avoidance-related perceived control rather than perceived control over PES use itself. Accordingly, its behavioral correspondence with the PES-use intention measure is imperfect within the TPB framework. Because both avoidance-related PBC and resistance self-efficacy concern participants’ perceived capacity to refrain from PES use, the two constructs also show conceptual and item-level overlap. They were retained as theoretically distinct measures in the present analyses, but their empirical interpretation should therefore not assume complete construct separation.

### Supplementary variables

Two additional variables were examined as theoretically informed supplementary variables. PES-related knowledge was operationalized as factual understanding of PES effects and risks. Although greater knowledge is often assumed to discourage PES use, findings remain mixed, suggesting that knowledge alone may be insufficient in the presence of motivational or normative pressures [6, 11, 20].

Risk acceptance was conceptualized as willingness to tolerate potential health risks in pursuit of short-term performance or appearance goals and has previously been associated with doping intentions and use [12, 21]. Knowledge and risk acceptance were treated as theoretically informed supplementary variables rather than components of a distinct theoretical model.

### Qualitative component

To capture prevention needs beyond the predefined theoretical constructs, 542 evaluable open-ended responses were analyzed using structured qualitative content analysis [22, 23]. The qualitative strand was intended to contextualize the quantitative findings and identify prevention-relevant themes extending beyond the predefined constructs [24–26].

Two exploratory research questions guided this component: (RQ1) Which thematic patterns characterize participants’ self-reported prevention needs? and (RQ2) How do these patterns align with TPB, HBM, and the supplementary variables, and where do they indicate additional dimensions beyond these models?

An overview of the study hypotheses and research questions is presented in Table 1.

**Table 1:** Overview of hypotheses and research questions

ID	Statement	Framework	Status
H1	More positive attitudes toward PES use → higher intention	TPB	Confirmatory (primary model)
H2	Stronger perceived social norms favoring PES use → higher intention	TPB	Confirmatory (primary model)
H3	H3: Lower avoidance-related perceived control → higher PES-use intention	TPB	Confirmatory (primary model)
H4	H4: Lower avoidance-related perceived control is associated with a higher likelihood of ever/current PES use	TPB	Confirmatory (primary model)
H5	Higher current PES-use intention is associated with a higher likelihood of ever/current PES use	TPB	Confirmatory (primary model)
H6	Higher risk perception → lower intention and lower likelihood of ever/current PES use	HBM	Complementary (theory-informed)
H7	Greater perceived benefits of PES use → higher intention	HBM	Complementary (theory-informed)
H8	Greater perceived barriers to abstaining from PES → higher intention	HBM	Complementary (theory-informed)
H9	Lower self-efficacy to resist PES → higher intention and higher likelihood of ever/current PES use	HBM	Complementary (theory-informed)
H10	Higher knowledge → lower intention and lower likelihood of ever/current PES use	Supplementary	Exploratory extension
H11	Higher risk acceptance → higher intention and higher likelihood of ever/current PES use	Supplementary	Exploratory extension
RQ1	Which thematic patterns characterize participants’ self-reported prevention needs?	Qualitative	Exploratory
RQ2	How do these patterns align with TPB, HBM, and supplementary variables?	Mixed methods	Exploratory

PBC was operationalized as avoidance-related perceived control, reflecting perceived control over avoiding or abstaining from PES rather than control over PES use itself. PBC and self-efficacy were reverse coded for the statistical analyses; higher analytical scores therefore represent lower perceived control over avoiding PES and lower self-efficacy to resist PES, respectively. Reverse coding affects score direction but not the behavioral target of the measures. Hypotheses concerning ever/current PES use refer to cross-sectional associations and do not imply prospective prediction or temporal ordering.

## Methods

### Study design

This study employed a cross-sectional mixed-methods design with a sequential explanatory analytical logic, integrating a quantitative survey with a qualitative content analysis of open-ended prevention responses. Quantitative and qualitative data were collected within the same survey wave, but the qualitative strand was analysed after the quantitative results (including the exploratory cluster analysis) to support interpretation and integration in a sequential explanatory logic. Mixed-methods approaches are particularly well-suited to capture both patterns of psychosocial factors and the contextual meanings associated with them [24, 25]. Following recommendations for the development of complex interventions [26], the qualitative strand was included to support interpretation of the quantitative findings. Although online surveys in health behavior research have faced critical discussion regarding representativeness and response quality [27, 28], multiple steps were undertaken to mitigate these concerns. The quantitative component examined constructs derived from the Theory of Planned Behavior (TPB) [14] and selected constructs from the Health Belief Model (HBM) [16], in addition to Knowledge regarding performance-enhancing substances (PES) and risk acceptance. The qualitative strand explored participants’ prevention needs, thereby providing contextual information beyond the quantitative models. Both strands were integrated through a joint display that cross-tabulated cluster membership with qualitative categories to examine convergence, divergence, and contextual relationships across the quantitative and qualitative findings [25]. Each construct was analyzed within its predefined theoretical framework to maintain conceptual clarity.

### Participants and procedure

A total of 772 recreational fitness practitioners (M_age = 28.9 years, SD = 8.7) were recruited in Germany. The present study uses the same underlying sample (*N* = 772) as Paasch et al. [29]; the overlap in data components and the distinct analytical focus of the present study are detailed in Supplementary Table S4. Eligibility criteria were: (a) age ≥ 18 years, (b) ≥ 2 weekly training sessions, and (c) informed consent for anonymous participation. Recruitment used convenience sampling in fitness centres and online communities—an established approach to reach hard-to-access populations [30]. Digital recruitment has recently been highlighted for its strengths and limitations in accessing gym-goers [31]. Although this limits representativeness, the approach increases feasibility in recreational sport settings. Given the sensitive nature of PES use, self-report data may also be subject to self-selection and social desirability biases, which are discussed under Limitations. The sample size exceeded conventional recommendations for the planned regression and cluster analyses [32]. Ethical approval was granted by the institutional review board (DHGS-EK-2025-006).

Participant characteristics are summarized in Table 2.

**Table 2:** Demographic and training characteristics of participants (N  = 772)

Variable	Category / Range	*n* (%) / M (SD)
Age (years)	—	28.9 (8.7)
Gender	Male	456 (59.1)
	Female	295 (38.2)
	Diverse	11 (1.4)
	No answer	10 (1.3)
Training frequency (per week)	—	3.1 (1.2)
Ever/current PES use	Yes	227 (29.4)
	No	545 (70.6)
Recruitment source	Gym	555 (71.9)
	Online community	217 (28.1)

PES = performance-enhancing substances; M = mean; SD = standard deviation.

### Quantitative measures

The multi-item measures used in the present study were operationalized specifically for the underlying survey and were informed by established theoretical frameworks and previous research rather than administered as verbatim reproductions of single previously published scales. TPB items were based on established measurement guidance for the Theory of Planned Behavior [14, 33], whereas the HBM-related items were informed by established Health Belief Model constructs [15, 16]. The knowledge and risk-acceptance items were informed by previous research on performance-enhancing substance use and doping-related decision-making [12, 34, 35]. Complete English-language item wording, response formats, coding procedures, and scoring information for all quantitative measures are provided in Supplementary Table S1. Because the present study focused on theory-based hypothesis testing rather than scale development or validation, full factor-analytic validation (EFA/CFA) was beyond its scope and is addressed as a limitation.

### TPB constructs (H1–H5)

TPB constructs were assessed using multi-item 7-point response scales with construct-specific anchors, consistent with established measurement guidelines [14, 33]. Attitude toward PES use was assessed with four items (α = 0.81), subjective norms with three items (α = 0.78), avoidance-related perceived behavioral control (PBC) with three items (α = 0.76), and intention to use PES with three items (α = 0.85). The PBC items were phrased in terms of participants’ perceived ability to avoid or abstain from PES use (e.g., ‘I could avoid using PES at any time if I wanted to’). Thus, the measure is more precisely characterized as avoidance-related perceived behavioral control rather than perceived control over PES use itself. For the analytical composite, these items were reverse coded; consequently, higher PBC scores in the statistical analyses represent lower perceived control over avoiding PES use. Reverse coding changed the direction of the analytical score but not the behavioral target of the underlying items. Because the intention items assessed future PES use (e.g., ‘I intend to use PES within the next 12 months’), behavioral correspondence between the PBC and intention measures was therefore imperfect within the TPB framework. Complete item wording, scoring procedures, and interpretation of higher scores are provided in Supplementary Table S1.

### HBM constructs (H6–H9)

Selected Health Belief Model constructs [15, 16] were assessed using 7-point Likert scales (1 = strongly disagree, 7 = strongly agree). Risk perception was assessed using a five-item composite capturing perceived health risks and severity of potential consequences associated with PES use (α = 0.79). Perceived benefits of PES use were assessed with three items (α = 0.80), perceived barriers to abstaining from PES with three items (α = 0.75), and self-efficacy to resist PES use with three items (α = 0.78). The original self-efficacy items assessed confidence in resisting or avoiding PES use, including under social or situational pressure. Given their shared focus on the perceived capacity to refrain from PES use, avoidance-related PBC and resistance self-efficacy show conceptual and item-level overlap. They were retained as theoretically distinct measures because they originated from different theoretical frameworks; however, given their conceptual and item-level overlap, their interpretation does not assume complete construct separation.

For the analytical composite, these items were reverse coded; higher analytical scores therefore represent lower self-efficacy to resist PES use. Complete item wording, scoring procedures, and interpretation of higher scores are reported in Supplementary Table S1.

### Supplementary variables (H10–H11)

PES-related knowledge was assessed with 10 true/false items (α = 0.72). Correct responses were summed to form a score ranging from 0 to 10, with higher scores indicating greater knowledge. Risk acceptance was assessed with four items (α = 0.83), with higher scores indicating greater willingness to accept health risks in pursuit of performance or appearance goals. Both constructs have been identified as relevant to PES-related decision-making [12, 34, 35]. Complete item wording and scoring information are provided in Supplementary Table S1.

### Behavioral outcomes

For each of six specified PES categories (AAS, growth hormone, TRT/testosterone, fat burners, stimulants, and boosters), participants indicated whether they had ever used or were currently using the respective substance. The categories “fat burners,” “stimulants,” and “boosters” were presented as broad substance categories without further product-level definitions. Accordingly, these categories may have encompassed heterogeneous products, including legally available stimulant-containing products such as caffeine-based pre-workout products. Responses across the six categories were collapsed into a binary indicator of ever/current PES use (1 = reported ever or current use of at least one category, *n* = 227, 29.4%; 0 = no reported ever or current use across all categories, *n* = 545, 70.6%). Because participants could report more than one substance category, category-specific frequencies were not mutually exclusive. Frequencies and prevalence estimates for each PES category are provided in Supplementary Table S2.

An overview of all study measures and their internal consistencies is presented in Table 3.

**Table 3:** Overview, scoring direction, and internal consistency of study measures

Construct	Items	Scoring interpretation	α
Attitude (TPB)	4	Higher = more favorable attitude toward PES use	0.81
Subjective norms (TPB)	3	Higher = stronger perceived social approval/normalization of PES use	0.78
Avoidance-related PBC (TPB)	3	Higher analytical score = lower perceived control over avoiding PES	0.76
Intention (TPB)	3	Higher = stronger PES-use intention	0.85
Risk perception (HBM)	5	Higher = greater perceived health risk	0.79
Perceived Benefits of PES Use (HBM)	3	Higher = greater perceived benefits of PES use	0.80
Perceived barriers (HBM)	3	Higher = greater perceived barriers to abstaining	0.75
Self-efficacy (HBM)	3	Higher analytical score = lower self-efficacy to resist PES	0.78
Knowledge	10	Higher = greater PES-related knowledge	0.72
Risk acceptance	4	Higher = greater risk acceptance	0.83

PES = performance-enhancing substances; PBC = perceived behavioral control; α = Cronbach’s alpha. PBC was operationalized as avoidance-related perceived control, reflecting perceived control over avoiding or abstaining from PES rather than control over PES use itself. PBC and self-efficacy items were reverse coded for the analytical composites; higher analytical scores therefore represent lower perceived control over avoiding PES and lower self-efficacy to resist PES, respectively. Reverse coding affected score direction but did not alter the behavioral target of the measures. Complete item wording, response formats, and scoring procedures are provided in Supplementary Table S1.

### Cluster analysis (Exploratory)

To identify exploratory subgroups of recreational fitness practitioners, cluster analysis was conducted using TPB constructs, HBM dimensions, knowledge, and risk acceptance. Prior to clustering, all clustering variables were z-standardized. Ward’s hierarchical clustering with squared Euclidean distance was first applied to examine the cluster structure and derive initial cluster centers, followed by k-means refinement to minimize within-cluster variance.

The three-cluster solution was retained based on the agglomeration pattern, interpretability, and cluster-size considerations. The largest relative increase in within-cluster heterogeneity occurred when moving from three to two clusters, indicating the merging of substantively distinct groups. A four-cluster solution yielded a comparatively small (*n* < 80) and substantively redundant additional cluster, whereas the three-cluster solution produced groups of *n* = 290, *n* = 250, and *n* = 232. However, the average silhouette coefficient was 0.181, indicating limited cluster separation [36, 37].

Accordingly, the three-cluster solution is treated as an exploratory and descriptive heuristic rather than as evidence of stable or validated participant types. Comparisons across the clustering variables are used descriptively to characterize the resulting profiles and are not interpreted as independent evidence of discriminant validity. Replication in independent samples using approaches such as latent profile analysis is needed to evaluate the robustness of these exploratory profiles.

### Qualitative data collection and analysis

An open-ended question asked: “What kind of prevention measures or support would help you or others in dealing with performance-enhancing substance use in the fitness scene?” A total of 542 participants provided evaluable responses. Responses were analyzed using structured qualitative content analysis [22, 23]. The coding framework was developed to capture prevention-relevant themes while allowing content extending beyond the predefined quantitative constructs to be represented.

Each response received one primary thematic assignment. Where a response contained additional distinct meaning-bearing statements, secondary thematic assignments were permitted; 87 respondents received at least one secondary assignment. Two independent coders coded the responses and achieved substantial interrater agreement before consensus (κ = 0.82). Disagreements were resolved through discussion and consensus. Frequencies reported in Supplementary Table S3 refer to the primary thematic assignments across the 542 respondents and therefore sum to *n* = 542; secondary assignments are not included in these percentages.

### Data integration

Quantitative and qualitative strands were integrated through a joint display cross-tabulating exploratory cluster membership with primary qualitative prevention themes. The joint display was used to examine convergence, divergence, and contextual relationships between the quantitative profiles and participants’ expressed prevention needs [24, 25]. Given the exploratory cluster solution and cross-sectional design, this integration was used for contextualization and hypothesis generation rather than to infer the effectiveness or appropriateness of specific prevention strategies for particular participant profiles.

### Statistical analyses

All statistical analyses followed established recommendations for quantitative psychology [32, 38]. Confirmatory analyses were conducted for hypotheses derived from the Theory of Planned Behavior (TPB), whereas Health Belief Model (HBM) constructs and the supplementary variables were evaluated as complementary and exploratory components. Results were interpreted with emphasis on effect sizes, confidence intervals, and the consistency of findings rather than on isolated p-values.

Analyses proceeded from descriptive to inferential procedures. Multiple linear regressions examined associations with current PES-use intention (H1–H3, H6–H11), whereas binary logistic regressions examined associations with ever/current PES use (H4–H11). Exploratory group comparisons were used descriptively to characterize the derived cluster profiles.

Effect sizes were reported as β, odds ratios with 95% CIs, Cohen’s d, and η² [39]. Significance was evaluated at α = 0.05 using two-tailed tests. Assumptions were checked (VIF < 5, residual normality, homoscedasticity). All analyses were conducted with SPSS 29.0. Item-level missingness was below 5%; among the 23 cases relevant to the difference in analytical N, missingness involved one item within a four-item scale. For four-item scales, composite scores were calculated as the mean of the available items when at least three of the four items (75%) had been completed; otherwise, the composite score was treated as missing. For the principal linear and logistic regression models, however, complete composite scores were available for all included predictors, and the ever/current PES-use outcome was complete because responses to the six PES-category items were required by the survey structure. Consequently, the effective analytical sample size for all principal regression models was *N* = 772, with no case loss due to listwise deletion. As a sensitivity analysis, multiple imputation was conducted using m = 5 imputed datasets. The imputation model included the scale items underlying Attitude, Subjective Norms, PBC, Intention, Risk Perception, Perceived Benefits, Barriers, Self-Efficacy, Knowledge, and Risk Acceptance; the six binary PES-category indicators; and age, gender, training frequency, and training duration. Pooled estimates were consistent with the primary analyses in coefficient direction and statistical significance, and no substantive conclusions changed. Formal adjustments for multiple testing were not applied, as analyses addressed distinct theoretical components and were interpreted in a theory-guided manner rather than as a single family of tests.

## Results

### Descriptive statistics and internal consistency

Table 4 presents descriptive statistics and internal consistency estimates for all multi-item constructs. Variables were computed as mean scores of their respective items on 7-point response scales, except knowledge, which was assessed as the sum of correct responses (range: 0–10). Inspection of distributions indicated no severe deviations from normality for the continuous study variables. Internal consistencies were acceptable to good across all scales.

**Table 4:** Descriptive statistics and internal consistency of study variables (N  = 772)

Variable	M	SD	α
Attitude (TPB)	3.93	0.84	0.81
Subjective Norms (TPB)	3.60	1.48	0.78
Avoidance-related Perceived Behavioral Control (TPB)	4.77	0.89	0.76
Intention (TPB)	4.34	0.83	0.85
Risk Perception (HBM)	4.52	0.82	0.79
Perceived Benefits of PES Use (HBM)	3.49	0.63	0.80
Perceived Barriers (HBM)	2.43	0.67	0.75
Self-Efficacy (HBM)	3.79	0.55	0.78
Knowledge (0–10 score)	4.22	2.50	0.72
Risk Acceptance	3.93	0.84	0.83

M = mean; SD = standard deviation; α = Cronbach’s alpha; TPB = Theory of Planned Behavior; HBM = Health Belief Model; PBC = perceived behavioral control. PBC was operationalized as avoidance-related perceived control. PBC and self-efficacy were reverse coded for the analytical composites; higher scores therefore represent lower perceived control over avoiding PES and lower self-efficacy to resist PES, respectively.

### Hypothesis testing by theoretical models

Theory of Planned Behavior (H1–H5).

### Results

The regression model examining associations of the three TPB constructs with current PES-use intention was significant, explaining 53.1% of the variance (adjusted R² = 0.529, F(3, 768) = 289.7, *p* < .001).

Attitude was not significantly associated with current PES-use intention (β = 0.00, *p* = .844; H1 not supported), whereas subjective norms showed a significant positive association (β = 0.41, *p* < .001; H2 supported). Avoidance-related PBC was also significantly associated with intention (β = 0.29, *p* < .001; H3 supported); given the reverse coding of the analytical PBC composite, this positive coefficient indicates that lower perceived control over avoiding PES was associated with higher PES-use intention.

The logistic regression model examining associations of the TPB constructs and current intention with ever/current PES use was not statistically significant, χ²(4) = 1.82, *p* = .769, with negligible explained variance (Nagelkerke R² = 0.003; effective *N* = 772). Avoidance-related PBC was not significantly associated with ever/current PES use (OR = 1.01, 95% CI [0.79, 1.28], *p* = .975; H4 not supported), and current PES-use intention was likewise not significantly associated with ever/current PES use (OR = 0.95, 95% CI [0.74, 1.21], *p* = .660; H5 not supported).

The results of the TPB hypothesis tests are summarized in Table 5.

**Table 5:** Theory of planned behavior: hypothesis tests

Hypothesis	Path	β / OR	95% CI	*p*	Supported
H1	Attitude → Current PES-use intention	0.00	—	0.844	No
H2	Subjective Norms → Current PES-use intention	0.41	—	< 0.001	Yes
H3	Avoidance-related PBCᵃ → Current PES-use intention	0.29	—	< 0.001	Yes
H4	Avoidance-related PBCᵃ → Ever/current PES use	1.01	[0.79, 1.28]	0.975	No
H5	Current PES-use intention → Ever/current PES use (OR)	0.95	[0.74, 1.21]	0.660	No

β = standardized regression coefficient; OR = odds ratio; CI = confidence interval; PBC = perceived behavioral control; PES = performance-enhancing substances. ᵃ PBC was operationalized as avoidance-related perceived control and reverse coded for the analytical composite; higher scores therefore represent lower perceived control over avoiding PES. Associations with ever/current PES use are cross-sectional and do not imply temporal prediction.

### Health belief model (H6–H9)

#### Results

The regression model examining associations of the four HBM-related constructs with current PES-use intention was significant, explaining 13.8% of the variance (adjusted R² = 0.135, F(4, 767) = 31.1, *p* < .001). Risk perception was not significantly associated with current PES-use intention (β = −0.04, *p* = .293; H6 not supported). Greater perceived benefits of PES use were significantly associated with higher intention (β = 0.24, *p* < .001; H7 supported), whereas perceived barriers to abstaining from PES were not significantly associated with intention (β = −0.06, *p* = .097; H8 not supported). Self-efficacy was significantly associated with intention (β = 0.21, *p* < .001; intention-related component of H9 supported); given the reverse coding of the analytical self-efficacy composite, this positive coefficient indicates that lower self-efficacy to resist PES was associated with higher PES-use intention.

The logistic regression examining associations of the four HBM-related constructs with ever/current PES use was not significant overall (Nagelkerke R² = 0.008, χ²(4) = 5.9, *p* = .206). None of the HBM-related constructs was significantly associated with ever/current PES use (all ps > 0.20). The results of the HBM hypothesis tests are summarized in Table 6.

**Table 6:** Health belief model (H6–H9)

Hypothesis	Predictor → Outcome	β / OR	95% CI	*p*	Supported
H6	Risk Perception → Current PES-use intention	−0.04	—	0.293	No
H7	Perceived Benefits of PES Use → Current PES-use intention	0.24	—	< 0.001	Yes
H8	Perceived Barriers to Abstaining → Current PES-use intention	−0.06	—	0.097	No
H9	Self-Efficacyᵃ → Current PES-use intention	0.21	—	< 0.001	Yes
H6 (Beh.)	Risk Perception → Ever/current PES use	0.95	[0.73, 1.24]	0.709	No
H7 (Beh.)	Perceived Benefits of PES Use → Ever/current PES use	1.10	[0.86, 1.41]	0.450	No
H8 (Beh.)	Perceived Barriers to Abstaining → Ever/current PES use	0.89	[0.70, 1.13]	0.349	No
H9 (Beh.)	Self-Efficacyᵃ → Ever/current PES use	1.11	[0.88, 1.40]	0.374	No

β = standardized regression coefficient; OR = odds ratio; CI = confidence interval; PES = performance-enhancing substances. ᵃ Self-efficacy was reverse coded for the analytical composite; higher scores represent lower self-efficacy to resist PES. Associations with ever/current PES use are cross-sectional and do not imply temporal prediction.

### Supplementary variables (H10–H11)

#### Results

The regression model examining associations of knowledge and risk acceptance with current PES-use intention was significant, explaining 8.5% of the variance (adjusted R² = 0.083, F(2, 769) = 36.0, *p* < .001). Greater PES-related knowledge was significantly associated with lower intention (β = −0.13, *p* < .001; intention-related component of H10 supported), whereas greater risk acceptance was significantly associated with higher intention (β = 0.28, *p* < .001; intention-related component of H11 supported).

The logistic regression examining associations of knowledge and risk acceptance with ever/current PES use was not significant overall (Nagelkerke R² = 0.006, χ²(2) = 4.5, *p* = .105). Neither knowledge (OR = 0.86, 95% CI [0.67, 1.10], *p* = .230) nor risk acceptance (OR = 1.15, 95% CI [0.92, 1.43], *p* = .216) was significantly associated with ever/current PES use.

The results of the analyses for the supplementary variables are summarized in Table 7.

**Table 7:** Supplementary variables (H10–H11)

Hypothesis	Predictor → Outcome	β / OR	95% CI	*p*	Supported
H10	Knowledge → Current PES-use intention	−0.13	—	< 0.001	Yes
H11	Risk Acceptance → Current PES-use intention	0.28	—	< 0.001	Yes
H10 (Beh.)	Knowledge → Ever/current PES use	0.86	[0.67, 1.10]	0.230	No
H11 (Beh.)	Risk Acceptance → Ever/current PES use	1.15	[0.92, 1.43]	0.216	No

β = standardized regression coefficient; OR = odds ratio; CI = confidence interval; PES = performance-enhancing substances. Associations with ever/current PES use are cross-sectional and do not imply temporal prediction.

Seven primary themes of prevention needs were identified, reflecting recurring patterns related to information, communication, practical support, and institutional responsibility. These themes represent categories of participants’ expressed prevention preferences rather than psychological or behavioral respondent types. An overview of the seven themes and their defining characteristics is presented in Table 8.

**Table 8:** Primary themes of self-reported prevention needs (n  = 542)

Theme	Definition	*n*	%
Knowledge Seekers	Preference for factual, accessible information	141	26.0
Trust Driven	Preference for credible professionals and experts	98	18.1
Pragmatists	Preference for concrete, gym-based practical measures	84	15.5
Dialogue Oriented	Preference for peer exchange and workshops	76	14.0
Emotionally Resistant	Preference for positive framing and rejection of fear appeals	57	10.5
Skeptics	Low acceptance of prevention approaches	49	9.0
Prevention Supporters	Preference for structural and policy-level interventions	37	6.8
Total		542	100.0

Frequencies and percentages refer to primary thematic assignments among the 542 participants who provided evaluable open-ended responses. Each respondent received one primary thematic assignment. Where responses contained additional distinct meaning-bearing statements, secondary thematic assignments were permitted; 87 respondents received at least one secondary assignment. Secondary assignments are not included in the frequencies and percentages reported in this table.

Taken together, these themes illustrate the heterogeneity of self-reported prevention preferences among recreational gym users and provide the basis for the exploratory mixed-methods integration presented in the subsequent joint display.

### Cluster analysis (Exploratory)

#### Procedure

To explore heterogeneity in psychosocial profiles, an exploratory cluster analysis was conducted based on TPB constructs (attitude, subjective norms, avoidance-related perceived behavioral control, intention), HBM-related constructs (risk perception, perceived benefits of PES use, perceived barriers, self-efficacy), knowledge, and risk acceptance. Ward’s hierarchical clustering with squared Euclidean distance was first applied to examine the cluster structure and derive initial cluster centers, followed by k-means refinement.

#### Solution

A three-cluster solution was retained based on the agglomeration pattern, interpretability, and cluster-size considerations. The largest relative increase in within-cluster heterogeneity occurred when moving from three to two clusters, indicating the merging of substantively distinct groups. A four-cluster solution yielded a comparatively small (*n* < 80) and substantively redundant additional cluster, whereas the three-cluster solution produced groups of *n* = 290, *n* = 250, and *n* = 232. The average silhouette coefficient was 0.181, indicating limited cluster separation. Accordingly, the three-cluster solution is treated as an exploratory and descriptive heuristic rather than as evidence of stable or validated participant types. Comparisons across the clustering variables are used descriptively to characterize the profiles and are not interpreted as independent evidence of discriminant validity. Cluster profiles (standardized means, descriptive patterns):

- Cluster 1: Norm-sensitive avoiders (*n* = 290; 38%).

Characterized by high subjective norms and elevated risk perception, combined with relatively low risk acceptance. Members reported comparatively low PES-use intention.

- Cluster 2: Ambivalent pragmatists (*n* = 250; 32%).

Characterized by moderate levels across most clustering variables, including knowledge and risk acceptance. PES-use intention was close to the sample mean.

- Cluster 3: Risk-accepting users (*n* = 232; 30%).

Characterized by high risk acceptance and low risk perception. This profile showed the highest PES-use intention and an above-average proportion of self-reported ever/current PES use.

### Interpretation

These profiles illustrate how combinations of theoretical constructs co-occur descriptively within the sample. Given the limited cluster separation, they should not be interpreted as stable participant types. Rather, they provide an exploratory description of psychosocial heterogeneity among recreational gym users.

The descriptive characteristics of the three cluster profiles are summarized in Table 9.

**Table 9:** Cluster profiles based on TPB, HBM, and supplementary variables (N  = 772)

Variable / Construct	Cluster 1: Norm-sensitive avoiders (*n* = 290; 38%)	Cluster 2: Ambivalent pragmatists (*n* = 250; 32%)	Cluster 3: Risk-accepting users (*n* = 232; 30%)
Subjective Norms (TPB)	High (+)	Moderate (≈ M)	Low (−)
Risk Perception (HBM)	High (+)	Moderate (≈ M)	Low (−)
Risk Acceptance	Low (−)	Moderate (≈ M)	High (+)
Intention (TPB)	Low (−)	Around sample mean	High (+)
Knowledge	Moderate	Moderate	Slightly below average (−)
Ever/current PES use	Below average	Around sample mean	Above average (+)
Overall descriptive profile	Norm-sensitive, lower-intention profile	Moderate, ambivalent profile	Higher risk acceptance and PES-use intention

Signs (+/−) indicate direction relative to the total sample mean (M). Cluster labels are heuristic and used for descriptive purposes only. The three-cluster solution showed limited separation (average silhouette = 0.181) and should therefore not be interpreted as evidence of stable or validated participant types.

### Integration of quantitative and qualitative findings

The joint display integrates the quantitative findings with the qualitative prevention themes and exploratory cluster profiles to examine convergence, divergence, and complementary patterns across the quantitative and qualitative strands. Given the cross-sectional design and limited cluster separation, the integrated findings are interpreted descriptively and as hypothesis-generating.

### Convergences

Subjective norms showed the strongest association with current PES-use intention (β = 0.41, *p* < .001), while qualitative responses highlighted peer exchange, trusted communicators, and gym-based practical measures as prevention-relevant themes. Greater PES-related knowledge was associated with lower intention (β = −0.13, *p* < .001), alongside participants’ expressed preference for accessible and evidence-based information. Risk acceptance was associated with higher intention (β = 0.28, *p* < .001), while qualitative responses also reflected variation in openness toward prevention and risk-oriented messaging. These convergences provide contextual support for the relevance of social, informational, and risk-related dimensions without implying causal relationships.

### Divergences

Risk perception was not significantly associated with current PES-use intention (β = −0.04, *p* = .293), whereas qualitative responses included both requests for risk-related information and rejection of fear-based messaging. Participants also emphasized communicator credibility, an aspect not directly represented in the quantitative TPB or HBM-related constructs. The exploratory cluster profiles further illustrated heterogeneity in combinations of psychosocial characteristics, but their limited separation precludes conclusions about profile-specific intervention effectiveness.

Overall, the integrated findings suggest areas of convergence between the quantitative and qualitative strands while also identifying complementary themes beyond the predefined theoretical constructs.

An integrated overview of the quantitative findings, qualitative themes, and cluster profiles is presented in Table 10.

**Table 10:** Joint display of quantitative findings, qualitative themes, and exploratory cluster profiles

Quantitative Finding / Profile	Qualitative Theme	Exploratory Integrated Interpretation
Subjective Norms → Intention (β = 0.41, *p* < .001)	Peer exchange and preference for credible communicators	Social and interpersonal factors were reflected across both strands and may warrant further investigation in prevention research.
Avoidance-related PBC → Intention (β = 0.29, *p* < .001)ᵃ	Requests for practical coping strategies and support	Lower perceived control over avoiding PES was associated with higher intention, while qualitative responses highlighted practical support as prevention relevant.
Risk Perception → Intention (n.s.) / Ever/current PES use (n.s.)	Requests for risk information alongside rejection of fear appeals	Quantitative associations were not significant, whereas qualitative responses indicated heterogeneous preferences regarding risk communication.
Knowledge → Intention (β = −0.13, *p* < .001)	Preference for transparent, evidence-based information	Greater knowledge was associated with lower intention, while information quality emerged as a prominent qualitative theme.
Risk Acceptance → Intention (β = 0.28, *p* < .001)	Variation in openness toward prevention and risk messaging	Greater risk acceptance was associated with higher intention, while qualitative responses indicated differing orientations toward prevention.
Cluster 1: Norm-sensitive avoiders (38%)	Themes emphasizing supportive environments and social context	The profile and qualitative findings suggest potential relevance of social-contextual factors for future hypothesis testing.
Cluster 2: Ambivalent pragmatists (32%)	Preferences for practical information and dialogue	The profile provides an exploratory context for investigating practical and participatory prevention approaches.
Cluster 3: Risk-accepting users (30%)	Skepticism and heterogeneous prevention preferences	The profile may inform future research examining how prevention is perceived among participants characterized by greater risk acceptance.

Quantitative analyses were based on *N* = 772; qualitative findings were based on 542 evaluable open-ended responses. Cluster profiles are exploratory and showed limited separation (average silhouette = 0.181). Integrated interpretations are descriptive and hypothesis-generating and should not be interpreted as evidence for the effectiveness of specific prevention strategies. ᵃ PBC was operationalized as avoidance-related perceived control and reverse coded for the analytical composite; higher scores represent lower perceived control over avoiding PES.

## Discussion

This mixed-methods study examined psychosocial factors associated with performance-enhancing substance (PES) use intentions among recreational gym users by integrating the Theory of Planned Behavior (TPB), selected Health Belief Model (HBM) constructs, and two theoretically informed supplementary variables (knowledge and risk acceptance). Overall, the findings indicate that several social and self-regulatory factors showed stronger associations with PES-use intention in the present sample than the examined cognitive risk appraisals.

Within the TPB, subjective norms and lower perceived control over avoiding PES were most strongly associated with higher PES-use intention, whereas attitudes showed no independent association. Among the HBM-related constructs, greater perceived benefits of PES use and lower self-efficacy to resist PES were associated with higher intention. Greater PES-related knowledge was associated with lower intention, whereas higher risk acceptance was associated with greater intention. None of the examined psychosocial variables were significantly associated with the retrospective/concurrent ever/current PES-use indicator. Given the cross-sectional design and the temporal mismatch between current intention and an outcome combining lifetime and current use, this finding should not be interpreted as evidence of an intention–behavior gap.

The qualitative findings and exploratory cluster analysis further illustrated heterogeneity in self-reported prevention preferences and psychosocial profiles among recreational gym users, highlighting social norms, self-regulatory capacities, and contextual influences as potentially relevant dimensions for future prevention research.

### Comparison with previous literature

Our findings are broadly consistent with meta-analytic evidence highlighting subjective norms and perceived behavioral control (PBC) as relevant correlates of health-risk intentions [12, 40]. In the present study, however, PBC was operationalized specifically as avoidance-related perceived control, and the observed association should therefore be interpreted as reflecting lower perceived control over avoiding PES rather than perceived control over PES use itself. In the present sample, attitudes were not independently associated with PES-use intention, whereas subjective norms showed a substantially stronger association. This pattern may be compatible with previous research emphasizing the relevance of peer norms and social context in recreational fitness settings [2, 6, 41]. However, broader normalization processes were not comprehensively assessed in the present study, and their role therefore remains an interpretation requiring further investigation.

Complementing the TPB findings, the HBM-related analyses indicated that greater perceived benefits of PES use were associated with higher PES-use intention, whereas lower self-efficacy to resist PES was likewise associated with higher intention. Risk perception was not significantly associated with intention. Qualitative responses additionally indicated heterogeneous preferences regarding risk communication, including resistance to fear-based messages and preferences for more positively framed or credible information. These qualitative findings provide contextual information but should not be interpreted as evidence that a particular communication strategy is more effective.

The supplementary variables provided additional explanatory context. Consistent with previous research [42], greater PES-related knowledge was associated with lower intention, while qualitative responses frequently emphasized accessible and evidence-based information. Conversely, greater risk acceptance was associated with higher intention [12]. These findings suggest that informational and risk-related orientations are associated with variation in PES-use intentions, although their temporal role cannot be determined from the present cross-sectional data.

Internationally, the prominence of subjective norms relative to attitudes is broadly compatible with findings reported across different recreational and performance-enhancement contexts [1]. The exploratory cluster analysis further illustrated that psychosocial characteristics may co-occur in different descriptive patterns. Although the present cluster solution differs conceptually from our previous person-centered analysis, both analyses were based on the same underlying sample and indicate descriptive heterogeneity among recreational gym users [29]. Given the limited separation of the present clusters, these similarities should not be interpreted as replication or validation of stable participant types.

### Theoretical implications

The present findings are broadly consistent with the Theory of Planned Behavior (TPB) as a useful framework for examining PES-related intentions in recreational fitness settings, while selected Health Belief Model (HBM) constructs provided complementary information on health-related evaluations and self-regulatory perceptions. The TPB model accounted for a substantial proportion of variance in current PES-use intention, whereas the HBM-related model explained a smaller proportion. By contrast, none of the examined psychosocial variables was significantly associated with the retrospective/concurrent ever/current PES-use indicator. Because current intention and ever/current use do not represent temporally aligned measures of prospective intention and subsequent behavior, these findings do not provide a direct test of the intention–behavior gap [43, 44].

The findings further suggest that TPB and selected HBM-related constructs capture partly complementary aspects of PES-use intention. Within the TPB, subjective norms and lower perceived control over avoiding PES were associated with higher intention. Among the HBM-related constructs, greater perceived benefits of PES use and lower self-efficacy to resist PES were associated with higher intention, whereas risk perception and perceived barriers were not significantly associated with intention. This pattern suggests that social influence, avoidance-related perceived control, perceived benefits, and resistance self-efficacy may be more closely related to current PES-use intention in this sample than general health-risk appraisal. However, the relative importance and temporal ordering of these constructs require longitudinal or experimental examination.

The qualitative findings concerning credibility, trust, and communication style, together with the exploratory cluster profiles, extend the quantitative results by highlighting broader social and contextual dimensions not fully represented by the predefined constructs. These findings suggest that future theoretical work may benefit from considering social influence, contextual opportunities, communication credibility, and self-regulatory capacity alongside intention-based models.

Taken together, the present findings indicate that intention-based models offer a useful framework for examining current PES-use intentions but cannot, on the basis of the present design, be evaluated as prospective explanations of subsequent PES use. The absence of significant associations with the ever/current use indicator should therefore be interpreted cautiously rather than as evidence against the theoretical relevance of intention. Future longitudinal research with temporally aligned measures of intention and subsequent behavior is needed to examine how social influence, self-regulatory processes, risk-related evaluations, and contextual conditions contribute to PES-related decision-making over time.

### Practical implications

Four theory-informed and practice-oriented implications may be considered as hypotheses for future prevention development rather than as empirically established intervention recommendations. First, the strong association between subjective norms and PES-use intention suggests that social and normative contexts warrant particular attention. Prevention approaches could therefore examine whether credible role models, peer communication, or visible substance-free training norms are acceptable and potentially useful within gym environments. The qualitative emphasis on peer exchange and credible communicators provides additional contextual support for investigating such approaches. Their effectiveness, however, cannot be inferred from the present study and requires prospective evaluation.

Second, the association between lower perceived control over avoiding PES and higher intention, together with the corresponding pattern for lower resistance self-efficacy, suggests that self-regulatory skills may represent a relevant target for future prevention research. Potential approaches include refusal-skills training, scenario-based exercises, or brief motivational interventions designed to strengthen perceived capacity to manage offers and social pressure. Whether such approaches reduce PES-use intentions or subsequent use in recreational fitness settings remains to be tested experimentally.

Third, the association between greater PES-related knowledge and lower intention, together with participants’ preference for credible and accessible information, suggests that information quality and communicator credibility warrant consideration in prevention development. Trainers, physicians, or other trusted professionals may represent plausible communication channels, although their comparative effectiveness was not examined here. Qualitative resistance to fear-based messaging further suggests that risk communication should be evaluated carefully. The Extended Parallel Process Model (EPPM) [45, 46] offers one theoretical framework for testing whether combinations of threat and efficacy influence acceptance or defensive responses to PES-related health communication.

Fourth, environmental approaches such as nudging may provide an additional avenue for future prevention research [47, 48]. Examples could include transparent supplement labeling, visible health-oriented social norms, or choice architectures that facilitate informed decisions. Given the association between risk acceptance and intention, future studies could examine whether responses to such approaches vary according to risk-related orientations. The present findings do not establish whether nudging, motivational approaches, or harm-reduction strategies are effective for particular participant profiles.

Taken together, these theory-informed implications are broadly compatible with international recommendations emphasizing evidence-based and context-sensitive doping prevention. However, their feasibility and effectiveness in recreational fitness settings require direct empirical evaluation. Future pilot and intervention studies could examine social-norm approaches, self-regulatory skills, credible risk communication, and environmental strategies individually and in combination. Beyond the exploratory quantitative profiles, the qualitative themes, including preferences for factual information, credible communicators, dialogue, and structural support, may help inform the design and acceptability testing of such interventions.

From a broader prevention perspective, previous research has also highlighted the potential relevance of multiple stakeholder groups, including trainers, gym operators, healthcare professionals, and supplement retailers. Such perspectives may complement the participant-centered findings of the present study by drawing attention to professional and structural conditions surrounding PES-related decisions. Value-based or morally focused approaches have likewise shown potential in previous doping-prevention research [49]. These approaches were not evaluated in the present study and should therefore be regarded as directions for future intervention research.

### Limitations

This study has several limitations that should be considered when interpreting the findings. First, the cross-sectional design precludes causal or temporal inferences regarding the relationships between psychosocial constructs, current PES-use intentions, and ever/current PES use. In particular, the retrospective/concurrent ever/current use indicator is not temporally aligned with current intention and therefore does not permit a prospective test of intention–behavior relationships. Second, the reliance on self-reported data may have led to underreporting of PES use due to social desirability or recall bias. Although data cleaning procedures, reliability checks, and plausibility screenings were applied, response bias cannot be fully excluded. In addition, recruitment via convenience sampling and voluntary participation may have introduced self-selection effects, thereby limiting representativeness and generalizability.

A separate limitation relates to the operationalization of the ever/current PES-use indicator. The categories “fat burners,” “stimulants,” and “boosters” were presented as broad substance categories without further product-level definitions and may therefore have been interpreted heterogeneously by participants. In particular, the inclusion of legally available stimulant-containing products, such as caffeine-based pre-workout products, cannot be ruled out. Consequently, the 29.4% prevalence of ever/current PES use should be interpreted as a broad composite indicator across the six assessed categories rather than as an estimate of illicit PES use. Future research should distinguish more clearly between specific substance classes and their legal or regulatory status when estimating PES-use prevalence.

A further limitation concerns measurement. Although the study measures were theory-informed and showed acceptable internal consistency, full construct validation using confirmatory factor analysis was beyond the scope of the present study. Importantly, PBC was operationalized as avoidance-related perceived control, with items assessing participants’ perceived ability to avoid or abstain from PES rather than perceived control over PES use itself. Although the PBC composite was reverse coded for analytical consistency, reverse coding changed only the direction of the score and not the behavioral target of the underlying items. Consequently, behavioral correspondence between the PBC measure and the PES-use intention measure was imperfect within the TPB framework. Moreover, avoidance-related PBC and resistance self-efficacy share a conceptual and item-level focus on participants’ perceived capacity to refrain from PES use, limiting assumptions of complete construct separation. These measurement characteristics should be considered when interpreting their respective associations with PES-use intention. Future research should use behaviorally matched TPB measures and further examine the factorial and discriminant validity of PBC and resistance self-efficacy in recreational fitness populations.

Third, the cluster analysis yielded exploratory and heuristic profiles that should not be interpreted as stable or validated participant types. The average silhouette coefficient (0.181) indicated limited separation between clusters, and comparisons across the variables used to derive the clusters cannot provide independent evidence of discriminant validity. Accordingly, the cluster solution should be understood as a descriptive structuring of psychosocial heterogeneity within the present sample. The present study uses the same underlying sample (*N* = 772) as Paasch et al. [29], and there is overlap in the basic survey instrument and raw data underlying the clustering indicators. However, the analytical focus differs: whereas the previous study primarily examined person-centered prevention segmentation and target-group identification, the present study focuses primarily on theory-based associations among TPB, HBM-related, knowledge, and risk-acceptance constructs and integrates these analyses with qualitative prevention themes. Similarities between the respective cluster-based patterns should therefore not be interpreted as independent replication or validation.

Replication using independent samples and cross-validation with person-centered approaches such as latent profile analysis or resampling procedures will be important for evaluating the stability and generalizability of these exploratory profiles.

Finally, although the Theory of Planned Behavior (TPB), selected Health Belief Model (HBM) constructs, and the supplementary variables capture several psychosocial dimensions potentially relevant to PES-related intentions, they do not fully encompass broader cultural, emotional, identity-related, and structural processes that may be associated with PES-related decisions in recreational fitness contexts. Future research integrating multiple levels of analysis may therefore provide a more comprehensive account of performance-enhancing substance use in recreational fitness settings.

### Future directions

Future research should pursue several complementary directions. First, longitudinal designs with temporally aligned assessments of intention and subsequent PES use are needed to examine prospective relationships between psychosocial constructs and behavior. Such designs would allow a more direct test of whether and under which conditions PES-use intentions translate into subsequent behavior and how social, self-regulatory, and contextual factors may shape this process.

Second, experimental and intervention-based studies should evaluate the feasibility and effectiveness of theory-informed prevention approaches, including normative communication, self-regulatory skills training, credible risk communication, and environmental strategies such as nudging. Initial evidence from theory-based prevention research in recreational fitness suggests that psychosocial factors and PES-related outcomes may be modifiable, although further randomized and longitudinal evaluation is required [50]. Cross-national research could additionally examine whether the prominence of social norms observed in the present sample generalizes across cultural and gym-based contexts.

Third, the exploratory cluster profiles require replication in independent samples using person-centered approaches such as latent profile analysis and appropriate validation procedures. Given the limited separation observed in the present study, future research should examine whether similar psychosocial patterns emerge independently and whether they provide incremental value beyond variable-centered models. Where feasible, triangulation of self-reported PES use with additional behavioral or biological indicators could further strengthen outcome assessment.

Fourth, further qualitative and mixed-methods research could examine how gym users, trainers, healthcare professionals, gym operators, and other relevant stakeholders understand PES-related risks, prevention needs, and communication. Such work may help clarify contextual and structural dimensions that are not fully captured by TPB or HBM-related constructs. Future theoretical work may also consider complementary frameworks such as the Prototype Willingness Model [51], particularly for examining less deliberative pathways to risk-related behavior.

Overall, future research should move from the present cross-sectional and hypothesis-generating findings toward longitudinal, experimental, and independently replicated designs capable of testing temporal processes, intervention effects, and the robustness of exploratory psychosocial profiles.

## Conclusion

This study highlights the relevance of psychosocial factors for understanding PES-use intentions among recreational gym users. Subjective norms, perceived control over avoiding PES, perceived benefits of PES use, resistance self-efficacy, knowledge, and risk acceptance were associated with intention, whereas none of the examined psychosocial variables was significantly associated with ever/current PES use. Qualitative findings further indicated heterogeneous prevention preferences, particularly regarding credible information, social context, and practical support. Given the cross-sectional design and exploratory cluster analysis, these findings should be interpreted as hypothesis-generating. Longitudinal and intervention-based research is needed to examine temporal relationships and evaluate whether these psychosocial and contextual factors can inform effective prevention.

## Supplementary information

Below is the link to the electronic supplementary material.


Supplementary Material 1


## Data Availability

The datasets generated and/or analysed during the current study are available from the corresponding author on reasonable request.

## Abbreviations

AAS: Anabolic-androgenic steroids
GH: Growth hormone
HBM: Health Belief Model
IPEDs: Image- and performance-enhancing drugs
PBC: Perceived behavioral control
PES: Performance-enhancing substances
TPB: Theory of Planned Behavior
TRT: Testosterone replacement therapy
