# Supplementary material for: Psychosocial correlates of performance-enhancing substance use intentions and prevention needs among recreational gym users: a theory-informed mixed-methods study
Source: BMC Psychol. 2026 Aug 29;14:1266. doi: 10.1186/s40359-026-05476-4 (PMC13525683; doi:10.1186/s40359-026-05476-4)
Supplement: Supplementary file 1 [file 40359_2026_5476_MOESM1_ESM.docx]

Supplementary Table S1

*Complete Item Wording, Response Formats, Scoring Procedures, and Interpretation of Quantitative Constructs*

| Construct | Item | Complete item wording | Scale / Response anchors | Correct answer | Coding | Higher analytical value means |
| --- | --- | --- | --- | --- | --- | --- |
| Attitude | ATT1 | Using PES is bad – good. | 1 = bad; 7 = good | — | Unchanged | More favorable attitude toward PES use |
|  | ATT2 | Using PES is harmful – beneficial. | 1 = harmful; 7 = beneficial | — | Unchanged | More favorable attitude toward PES use |
|  | ATT3 | Using PES is morally unacceptable – morally acceptable. | 1 = morally unacceptable; 7 = morally acceptable | — | Unchanged | More favorable attitude toward PES use |
|  | ATT4 | Using PES is ineffective – effective. | 1 = ineffective; 7 = effective | — | Unchanged | More favorable attitude toward PES use |
| Subjective Norms | SN1 | People who are important to me would approve if I used PES. | 1 = strongly disagree; 7 = strongly agree | — | Unchanged | Greater perceived social approval |
|  | SN2 | My friends would accept it if I used PES. | 1 = strongly disagree; 7 = strongly agree | — | Unchanged | Greater perceived social approval |
|  | SN3 | PES use is accepted/common within my social or training environment. | 1 = strongly disagree; 7 = strongly agree | — | Unchanged | Greater perceived normalization |
| Perceived Behavioral Control (PBC) | PBC1 | I could avoid using PES at any time if I wanted to. | 1 = strongly disagree; 7 = strongly agree | — | Reverse coded: 8 − original response | Lower perceived control over avoiding PES |
|  | PBC2 | If I wanted to, I could easily abstain from PES. | 1 = strongly disagree; 7 = strongly agree | — | Reverse coded: 8 − original response | Lower perceived control over avoiding PES |
|  | PBC3 | It would be easy for me to avoid PES even if others around me used them. | 1 = strongly disagree; 7 = strongly agree | — | Reverse coded: 8 − original response | Lower perceived control over avoiding PES |
| Intention | INT1 | I intend to use PES within the next 12 months. | 1 = strongly disagree; 7 = strongly agree | — | Unchanged | Stronger PES-use intention |
|  | INT2 | It is likely that I will use PES in the future. | 1 = very unlikely; 7 = very likely | — | Unchanged | Stronger PES-use intention |
|  | INT3 | I plan to use PES in the coming months. | 1 = strongly disagree; 7 = strongly agree | — | Unchanged | Stronger PES-use intention |
| Risk Perception | RP1 | I consider PES use to be harmful to health. | 1 = strongly disagree; 7 = strongly agree | — | Unchanged | Greater perceived risk |
|  | RP2 | The possible health consequences of PES use can be serious. | 1 = strongly disagree; 7 = strongly agree | — | Unchanged | Greater perceived severity |
|  | RP3 | I am aware of possible long term health consequences of PES. | 1 = strongly disagree; 7 = strongly agree | — | Unchanged | Greater perceived risk |
|  | RP4 | I see a high risk of psychological or physical consequences from PES use. | 1 = strongly disagree; 7 = strongly agree | — | Unchanged | Greater perceived risk |
|  | RP5 | Overall, taking PES poses a significant threat to a person’s long-term wellbeing. | 1 = strongly disagree; 7 = strongly agree | — | Unchanged | Greater perceived risk |
| Perceived Benefits of PES Use | BEN1 | PES could help me achieve my training goals more quickly. | 1 = strongly disagree; 7 = strongly agree | — | Unchanged | Greater perceived benefits of PES use |
|  | BEN2 | PES could give me advantages over others. | 1 = strongly disagree; 7 = strongly agree | — | Unchanged | Greater perceived benefits of PES use |
|  | BEN3 | PES could accelerate the physical or performance progress I want to achieve. | 1 = strongly disagree; 7 = strongly agree | — | Unchanged | Greater perceived benefits of PES use |
| Perceived Barriers to Abstaining | BAR1 | Avoiding PES would make it more difficult for me to achieve my desired training progress. | 1 = strongly disagree; 7 = strongly agree | — | Unchanged | Greater perceived barriers to abstaining |
|  | BAR2 | Social influences make it difficult for me to avoid PES. | 1 = strongly disagree; 7 = strongly agree | — | Unchanged | Greater perceived barriers to abstaining |
|  | BAR3 | Remaining PES free during challenging training periods requires considerable effort. | 1 = strongly disagree; 7 = strongly agree | — | Unchanged | Greater perceived barriers to abstaining |
| Self-Efficacy | SE1 | I am confident that I can refuse PES even when they are recommended to me. | 1 = not at all confident; 7 = completely confident | — | Reverse coded: 8 − original response | Lower self-efficacy to resist PES |
|  | SE2 | I feel capable of making progress without using PES. | 1 = not at all capable; 7 = completely capable | — | Reverse coded: 8 − original response | Lower self-efficacy to resist PES |
|  | SE3 | I am confident that I can abstain from PES even when people around me use them. | 1 = not at all confident; 7 = completely confident | — | Reverse coded: 8 − original response | Lower self-efficacy to resist PES |
| Risk Acceptance | RA1 | I am willing to accept health risks in order to make faster physical progress. | 1 = strongly disagree; 7 = strongly agree | — | Unchanged | Greater risk acceptance |
|  | RA2 | I would accept possible side effects if this promoted muscle growth. | 1 = strongly disagree; 7 = strongly agree | — | Unchanged | Greater risk acceptance |
|  | RA3 | I accept health risks if they allow me to reach my sporting goals more quickly. | 1 = strongly disagree; 7 = strongly agree | — | Unchanged | Greater risk acceptance |
|  | RA4 | I would use substances even if their long term health consequences were not known. | 1 = strongly disagree; 7 = strongly agree | — | Unchanged | Greater risk acceptance |
| Knowledge | K1 | Long-term use of anabolic steroids can suppress the body's natural testosterone production. | True / False / Don't know | True | Correct = 1; incorrect / Don't know = 0 | Greater factual PES knowledge |
|  | K2 | Using performance-enhancing substances carries no risk of cardiovascular side effects if taken in low doses. | True / False / Don't know | False | Correct = 1; incorrect / Don't know = 0 | Greater factual PES knowledge |
|  | K3 | Anabolic steroid use can cause severe liver damage and altered lipid profiles. | True / False / Don't know | True | Correct = 1; incorrect / Don't know = 0 | Greater factual PES knowledge |
|  | K4 | Psychological side effects such as mood swings or increased aggressiveness can occur during PES use. | True / False / Don't know | True | Correct = 1; incorrect / Don't know = 0 | Greater factual PES knowledge |
|  | K5 | Dietary supplements declared as "all-natural boosters" are guaranteed to be free of prohibited pharmaceutical substances. | True / False / Don't know | False | Correct = 1; incorrect / Don't know = 0 | Greater factual PES knowledge |
|  | K6 | Abruptly stopping anabolic steroid use without medical supervision can trigger depressive symptoms. | True / False / Don't know | True | Correct = 1; incorrect / Don't know = 0 | Greater factual PES knowledge |
|  | K7 | Exogenous human growth hormone (hGH) use does not affect glucose metabolism or insulin resistance. | True / False / Don't know | False | Correct = 1; incorrect / Don't know = 0 | Greater factual PES knowledge |
|  | K8 | High doses of caffeine-based stimulants and fat burners can cause severe cardiac arrhythmias. | True / False / Don't know | True | Correct = 1; incorrect / Don't know = 0 | Greater factual PES knowledge |
|  | K9 | Post-cycle therapy (PCT) completely eliminates all long-term health risks associated with anabolic steroid use. | True / False / Don't know | False | Correct = 1; incorrect / Don't know = 0 | Greater factual PES knowledge |
|  | K10 | Continuous use of anabolic steroids in men can lead to irreversible testicular atrophy. | True / False / Don't know | True | Correct = 1; incorrect / Don't know = 0 | Greater factual PES knowledge |

*Note.* PES = performance-enhancing substances; PBC = perceived behavioral control. Scale scores for the psychosocial constructs were calculated as item means. PBC and self-efficacy items were phrased in a protective direction but reverse coded for the analytical composites using 8 − original response. Accordingly, higher analytical scores represent lower perceived control over avoiding PES and lower self-efficacy to resist PES, respectively. Risk perception was calculated as the mean of five items. Knowledge items were scored as correct = 1 and incorrect or “Don't know” = 0 and summed to a total score ranging from 0 to 10; higher scores indicate greater factual PES knowledge.

PBC was operationalized as avoidance-related perceived control rather than perceived control over PES use itself. Accordingly, behavioral correspondence between the PBC and PES-use intention measures was imperfect. PBC and resistance self-efficacy also show conceptual and item-level overlap in their shared focus on participants’ perceived capacity to refrain from PES use and should therefore not be interpreted as completely distinct constructs.

Supplementary Table S2

*Prevalence of Assessed PES Categories*

| PES category | Included in composite | Assessment | n | % |
| --- | --- | --- | --- | --- |
| AAS | Yes | Ever/current | 42 | 5.4 |
| Growth hormone | Yes | Ever/current | 18 | 2.3 |
| TRT/testosterone | Yes | Ever/current | 35 | 4.5 |
| Fat burners | Yes | Ever/current | 89 | 11.5 |
| Stimulants | Yes | Ever/current | 112 | 14.5 |
| Boosters | Yes | Ever/current | 145 | 18.8 |
| Any defined PES | Yes | Ever/current | 227 | 29.4 |

*Note.* Category-specific frequencies are not mutually exclusive because participants could report use of more than one PES category. The categories “fat burners,” “stimulants,” and “boosters” were presented as broad substance categories without further product-level definitions and may therefore have encompassed heterogeneous products, including legally available stimulant-containing products such as caffeine-based pre-workout products. The binary ever/current PES-use indicator was coded as 1 when participants reported ever or current use of at least one of the six assessed categories and as 0 when no use was reported across all six categories. Accordingly, the 29.4% prevalence should be interpreted as a broad composite indicator across the six assessed categories rather than as an estimate of illicit PES use.

Supplementary Table S3

*Primary Themes of Self-Reported Prevention Needs*

| Theme | Definition | n | % |
| --- | --- | --- | --- |
| Knowledge Seekers | Factual and accessible information | 141 | 26.0 |
| Trust Driven | Credible professionals and experts | 98 | 18.1 |
| Pragmatists | Concrete gym-based practical measures | 84 | 15.5 |
| Dialogue Oriented | Peer exchange and workshops | 76 | 14.0 |
| Emotionally Resistant | Positive framing and rejection of fear appeals | 57 | 10.5 |
| Skeptics | Low prevention acceptance | 49 | 9.0 |
| Prevention Supporters | Structural and policy interventions | 37 | 6.8 |
| Total |  | 542 | 100.0 |

*Note.* Frequencies and percentages refer to primary thematic assignments among the 542 participants who provided evaluable open-ended responses. Each respondent received one primary thematic assignment. Where responses contained additional distinct meaning-bearing statements, secondary thematic assignments were permitted; 87 respondents received at least one secondary assignment. Secondary assignments are not included in the frequencies and percentages reported in this table.

**Supplementary Table S4**

*Comparison of Paasch et al. (2026a) and the Present Study Using the Shared Survey Sample*

| Dimension | Paasch et al. (2026a) | Present study |
| --- | --- | --- |
| Underlying sample | Same survey sample of N = 772 recreational gym users in Germany. | Same survey sample of N = 772 recreational gym users in Germany. |
| Primary focus | Person-centered prevention segmentation and identification of participant profiles. | Theory-specified analysis of psychosocial associations with current PES-use intention and ever/current PES use. |
| Primary research question | Which participant profiles can be identified with respect to psychosocial characteristics and prevention-related needs? | Which TPB constructs, selected HBM-related constructs, knowledge, and risk acceptance are associated with current PES-use intention and ever/current PES use, and how can qualitative prevention themes contextualize these associations? |
| Quantitative variables | Psychosocial indicators used primarily for person-centered segmentation and descriptive profile comparison. | TPB constructs, selected HBM-related constructs, knowledge, risk acceptance, current PES-use intention, and the binary ever/current PES-use indicator. |
| Principal quantitative analyses | Cluster analysis as the principal analytical approach, accompanied by descriptive and between-profile comparisons. | Multiple linear and logistic regression models as the principal hypothesis-testing analyses; cluster analysis retained only as an exploratory descriptive component. |
| Cluster component | Central to the person-centered segmentation framework. | Secondary and explicitly exploratory; overlapping cluster-based patterns are identified as deriving from the shared dataset and are not presented as independent replication or validation. |
| Qualitative component | Participant-reported prevention preferences examined in relation to prevention segmentation. | Open-ended prevention responses used to identify thematic categories and to contextualize the quantitative findings within the mixed-methods integration. |
| Mixed-methods role | Primarily supports person-centered prevention segmentation and target-group interpretation. | Primarily integrates theory-specified quantitative associations with participant-reported prevention themes; integration is descriptive and hypothesis-generating. |
| Behavioral outcome emphasis | PES-related profile characteristics and prevention-relevant segmentation. | Ever/current PES use defined as reported ever or current use of at least one of six assessed PES categories; analyzed as a cross-sectional behavioral indicator. |
| Interpretive emphasis | Segmentation of recreational gym users for prevention-oriented profile description. | Associations among theoretical constructs and PES-use intention, cautious interpretation of the ever/current use indicator, and generation of hypotheses for future prevention research. |
| Relationship between publications | Earlier analysis of the shared survey dataset with a primarily person-centered analytical focus. | Uses the same underlying sample and overlapping survey variables but addresses a distinct primary analytical question; overlapping analyses and data components are explicitly disclosed. |

*Note.* PES = performance-enhancing substances; TPB = Theory of Planned Behavior; HBM = Health Belief Model. Both publications use the same underlying survey sample (N = 772). The table is provided to make the areas of overlap and the distinct primary analytical purposes of the two publications explicit. Overlapping cluster-based and qualitative components in the present study are not interpreted as independent replication of findings from Paasch et al. (2026a).
